# Ten years later: An update on the status of collections of endemic Gulf of Mexico fishes put at risk by the 2010 Oil Spill

**DOI:** 10.3897/BDJ.12.e113399

**Published:** 2024-03-08

**Authors:** Prosanta Chakrabarty, Alec J. Sheehy, Xavier Clute, Shannon B. Cruz, Brandon Ballengée

**Affiliations:** 1 Louisiana State Unviersity, Museum of Natural Science, Baton Rouge, Louisiana, United States of America Louisiana State Unviersity, Museum of Natural Science Baton Rouge, Louisiana United States of America; 2 Department of Biological Sciences, Louisiana State University, Baton Rouge, Louisiana, United States of America Department of Biological Sciences, Louisiana State University Baton Rouge, Louisiana United States of America; 3 Canadian Museum of Nature, Zoology, Ottawa, Ontario, Canada Canadian Museum of Nature, Zoology Ottawa, Ontario Canada; 4 American Museum of Natural History, Division of Vertebrate Zoology, New York, New York, United States of America American Museum of Natural History, Division of Vertebrate Zoology New York, New York United States of America; 5 Smithsonian Institution, National Museum of Natural History, Department of Vertebrate Zoology, Washington, D.C., United States of America Smithsonian Institution, National Museum of Natural History, Department of Vertebrate Zoology Washington, D.C. United States of America; 6 Louisiana State University, Museum of Natural Science, Baton Rouge, LA, United States of America Louisiana State University, Museum of Natural Science Baton Rouge, LA United States of America; 7 Tulane University Biodiversity Research Institute, Belle Chasse, LA, United States of America Tulane University Biodiversity Research Institute Belle Chasse, LA United States of America; 8 Museum of Natural Science, Louisiana State University, Baton Rouge, LA, United States of America Museum of Natural Science, Louisiana State University Baton Rouge, LA United States of America; 9 Department of Ecology and Evolutionary Biology, Tulane University,, New Orleans, LA, United States of America Department of Ecology and Evolutionary Biology, Tulane University, New Orleans, LA United States of America

**Keywords:** conservation, Deepwater Horizon, ichthyofauna, Macando

## Abstract

The 2010 Gulf of Mexico Deepwater Horizon was the largest oil spill in human history that occurred during a 12-week period in a region less than 100 km from the coast of Louisiana; however, after more than a decade of post-spill research, few definitives can be said to be known about the long-term impacts on the development and distribution of fishes in and around the region of the disaster. Here, we examine endemic Gulf of Mexico fish species that may have been most impacted by noting their past distributions in the region of the spill and examining data of known collecting events and observations over the last twenty years (ten years prior to the spill, ten years post-spill). Five years post-spill, it was reported that 48 of the Gulf’s endemic fish species had not been collected and, with expanded methods, we now report that 29 (of the 78 endemic species) have not been reported in collections since 2010 (five of these are only known from observations post-spill). Although the good news that some previously ‘missing’ species have been found may be cause to celebrate, the lack of information for many species remains a cause for concern given focused sampling efforts post-spill.

## Introduction

The 2010 Gulf of Mexico Deepwater Horizon Oil Spill (DWH), MC 252 or Macondo blowout, was the largest accidental oil spill in history (Crone and Tolstoy 2010, Rabalais 2014, Murawski et al. 2023). The tremendous amount of oil spilled during DWH was estimated at 134 million gallons (down from greater than 200 million reported in earlier sources; or 507 million liters versus the 757 million liters reported earlier), which resulted in an immediate contamination area of 149,000 km and continued to spread through currents widely in the Gulf of Mexico (GOM) (Rabalais 2011, Ramseur and Hagerty 2014, MacDonald et al. 2015, Berenshtein et al. 2020). The spill lasted from the explosion and blowout on 20 April 2010 until the well was contained on 15 July 2010 (Petrolia 2014).

Coupled with the fact that it occurred in the deep sea (> 1000 m depth) and with the coordinated release of more than a million gallons of dispersant, which has been suggested to have made the DWH oil as much as 52% more toxic, more difficult to clean up and increased its impact to wildlife (Rico-Martínez et al. 2013). It has been suggested almost 100 million gallons (379 million liters) of DWH oil combined with dispersants remains in the Gulf and is one of the worst pollution events in history (Goodbody-Gringley et al. 2013, Ramseur and Hagerty 2014).

More than a decade after the end of the spill, the long-term effects of DWH are still not fully understood. Recent research has suggested that there have been some persistent ecological effects including damage to deep ocean coral communities, harm to oyster fisheries, loss of marshlands and population declines of marine mammals, sea turtles and seabirds (Barron et al. 2020).

Some fish species appear to have been particularly at risk and impacted by the 2010 Oil Spill with evidence of physical and developmental abnormalities reported and evidence of extirpations (Whitehead et al. 2012, Incardona et al. 2014, Dubansky et al. 2013, Brette et al. 2014, Mager et al. 2014, Alloy et al. 2016, Chakrabarty et al. 2016). Further, risks from oil production related polycyclic aromatic hydrocarbons (PAH) exposure and concentrations in fishes is widespread in the GOM and will likely continue as extraction of petrochemical intensifies (Turner and Rabalais 2019, Pulster et al. 2020). These include Taylor Energy’s MC20 oil spill which began in 2004 and continues today (Schrope 2013, Mason 2019, Schrope 2013). Linardich et al. (2019) reported that habitat degradation is the leading cause of risk to endemic GOM species and oil spills are certainly part of that degredation issue.

The Gulf of Mexico is one of the most biologically rich and resilient marine environments in the world with 1541 fish species known from the region, 78 of which are reported to be endemic to the Gulf (i.e. found only in the Gulf; McEachran (2009), Chakrabarty et al. (2016)). Although many commercially valuable fish species populations have been examined following DWH, most Gulf ichthyofauna as a whole have received little attention (Fodrie et al. 2014, Murawski et al. 2023). The IUCN has suggested that upwards of 25% of the Gulf’s endemic fishes may be threatened with extinction (Linardich et al. 2019, IUCN 2023). Chakrabarty et al. (Chakrabarty et al. 2016) examined museum records for the occurrence of all known endemic Gulf fish species (77 spp. thought to be endemic at the time) five years after the spill and reported that 48 species of fish had not been officially collected (i.e. vouchered in natural history collections) since the 2010 spill. Of these, 14 species were designated as being of ‘greatest concern’ as they may have been most impacted by DWH because of their past distributions being largely (> 35%) within the spill region.

In this current study, we re-examine museum records (2000-2020) of the 78 endemic species (adding the recently described American Pocket shark, *Mollisquamamississippiensis*, as a Gulf endemic) using data from The Global Biodiversity Information Facility (GBIF: https://www.gbif.org/), FishNet2 (http://www.fishnet2.net/) and recent literature.

## Material and methods

The documented occurrences of 78 species endemic to the Gulf of Mexico were tallied using two databases: The Global Biodiversity Information Facility (GBIF) and FishNet2. To complement data about voucher material, an extensive review of the literature on individual species was also performed to account for observations not involving collections. The International Union for Conservation of Nature’s Red List of Threatened Species (abbreviated “IUCN Red List” throughout) status was also reviewed and reported here when available (https://www.iucnredlist.org/).

GBIF recently changed their data algorithm to include observed species in addition to vouchered specimens. To accurately measure the change in species populations, the data from the total identified specimens (vouchered and observed in nature) from GBIF were graphed separately and compared to the total vouchered specimens from FishNet2 from 2000 to 2020. Only species found in the Gulf of Mexico and Caribbean Region were accounted for in the data, unless otherwise noted. We last gathered data from these websites on 20 April 2023.

A scatterplot graph was created in Microsoft Excel by plotting the Number of Occurrence(s) on the y-axis and the Number of Years on the x-axis. Collections data from GBIF are denoted as “GBIF Preserved” (orange circles) when a voucher specimen was collected, human observation data from GBIF are denoted as “GBIF Observed” (blue triangles) and collections data from FishNet2 are denoted by “FishNet2 Preserved” (grey diamonds) to indicate the type(s) of data that each database reported. No graph is included if species were not sampled between 2000-2020 (10 years before and 10 years after DWH).

Chakrabarty et al. (2016), Chakrabarty et al. (2012) and Chakrabarty et al. 2016 listed fish species of 'conservation concern' based on their known distribution within the spill zone or proximity to the DWH oil spill surface slick. Here, we refer to these as “Species of Greatest Concern” (if over 35% of their range was in the spill zone) or “Lesser Concern” (if less than 35% of their range was in the spill zone, but they are still considered endemic). No scatterplot is included for species lacking collections in the 20 year period of our survey, but we include information about the last observations/collections events when that is known.

Eschmeyer’s Catalog of Fishes (https://www.calacademy.org/scientists/projects/eschmeyers-catalog-of-fishes) was used to obtain current valid taxonomic names including the authority (authors of original description) and the family name (Fricke et al. 2023). Please note that following taxonomic convention, the taxonomic-authority reference following the scientific name is only presented in parentheses if the species was described in a different genus than it is currently in, otherwise there are no parentheses.

Species are presented using the taxonomy of Nelson et al. (2016), as well as the phylogenetic classification of Near and Thacker (2023) for Actinopterygii (species are listed in alphabetical order within each family and major clade, i.e. "Percomorpha").

## Results

AGNATHA: MYXINIFORMES (Hagfishes)

*Eptatretusminor* Fernholm and Hubbs, 1981 in Fernholm and Hubbs (1981); (Myxinidae). The species was last reported in 2009 off the coast of Mississippi. Status listed as “Data Deficient” on the IUCN Red List (Anonymous 2011).

*Eptatretusspringeri* (Bigelow and Schroeder, 1952) in Bigelow and Schroeder (1952); (Myxinidae), Gulf hagfish. Status listed as “Least Concern” on the IUCN Red List (Mincarone and Mok 2022).

Fig. 1

CHONDRICHTHYES (Elasmobranchs, cartilaginous fishes)

CARCHARHINIFORMES

*Mustelussinusmexicanus* Heemstra, 1997 in Heemstra (1997); (Triakidae), Gulf of Mexico smoothound. Status listed as “Least Concern” on the IUCN Red List (Carlson et al. 2021).

*Parmaturuscampechiensis* Springer, 1979 in Springer (1979); (Pentanchidae), Campeche catshark. Collected once in 1970 in the continental slope off Veracruz, Mexico. Status listed as “Least Concern” on the IUCN Red List (Kyne and Herman 2020).

RAJIFORMES

*Dipturusolseni* (Bigelow and Schroeder, 1951) in Bigelow and Schroeder (1951); (Rajidae), Spreadfin skate. The species was last reported in 2011 off the coast of Alabama. Status listed as “Least Concern” on the IUCN Red List (Kulka et al. 2020).

*Dipturusoregoni* (Bigelow and Schroeder, 1958) in Bigelow and Schroeder (1958); (Rajidae), Hooktail skate. Status listed as “Least Concern” on the IUCN Red List (Crysler et al. 2020).

*Leucorajalentiginosa* (Bigelow and Schroeder, 1951) in Bigelow and Schroeder (1951); (Rajidae), Freckle skate. Species last reported in GOM on FISHNET2 in 2000. *L.lentiginosa* in GBIF. Observed twice in 2012 off the western coast of Cozomel, Mexico by divers. Status listed as “Least Concern” on the IUCN Red List (Crysler et al. 2020).

*Rostrorajatexana* (Chandler, 1921) formerly *Rajatexana* in Chandler (1921); (Rajidae), Roundel skate. Status listed as “Least Concern” on the IUCN Red List (Dulvy et al. 2021).

*Springeriafolirostris* Bigelow and Schroeder, 1951 in Bigelow and Schroeder (1951) (formerly *Anacanthobatisfolirostris*); (Anacanthobatidae), Leafnose skate. Species was last collected in 2004 off the coast of Louisiana. Status listed as “Least Concern” on the IUCN Red List (Crysler et al. 2020).

SQUALIFORMES (sleeper sharks and dogfish)

*Etmopterusschultzi* Bigelow, Schroeder, and Springer, 1953 in Bigelow et al. (1953); (Etmopteridae), Fringefin lanternshark. The species was last reported in the GOM in 2009 off the coast of Alabama. Status listed as “Least Concern” on the IUCN Red List (Cotton et al. 2021).

*Mollisquamamississippiensis* Grace, Doosey, Denton, Naylor, Bart, Maisey, 2019 in Grace et al. 2019; (Dalatiidae), American Pocket shark. Species was collected once in 2010 prior to DWH in the central GOM. Status listed as “Least Concern” on the IUCN Red List (Kyne and Herman 2020).

Fig. 2

ACTINOPTERYGII (Ray-finned fishes)

HOLOSTEI

*Atractosteusspatula* (Lacepède, 1803) Lacepède (1803); (Lepisosteidae), Alligator gar; note that this species is represented here by the Gulf of Mexico samples and that this species is predominantly found in freshwaters whose collections are not shown here. Status listed as “Least concern” on the IUCN Red List (Collette et al. 2019).

*Lepisosteusoculatus* Winchell, 1864 in Winchell (1864); (Lepisosteidae), Spotted gar; note that this species is represented here by the Gulf of Mexico samples and that this species is predominantly found in freshwaters whose collections are not shown here. Status listed as “Least Concern” on the IUCN Red List (Collette et al. 2019).

Fig. 3

ELOPOMORPHA

Congridae

*Heterocongerluteolus* Smith, 1989 in Smith (1989); (Congridae), Yellow garden eel. *H.luteolus* has been GBIF observed seven times and vouchered eight times off the south-eastern coast of Florida since 2014, but not in the Gulf of Mexico since 2010. Status listed as “Least Concern” on the IUCN Red List (Smith 2015).

Muraenidae

*Monopenchelysacuta* (Parr, 1930) in Parr (1930); (Muraenidae), Redface Moray Eel. Species was last reported in the GOM in 2007 with the most recent report being 2010 in French Polynesia. Status listed as “Data Deficient” on the IUCN Red List (McCosker 2010).

Ophichthidae

*Gordiichthysergodes* McCosker, Böhlke and Böhlke, 1989 in McCosker et al. (1989); (Ophichthidae), Irksone eel. Species last reported on FishNet2 in 2006 off the Florida coast. Status listed as “Data Deficient” on the IUCN Red List (McCosker 2015).

*Gordiichthysleibyi* McCosker and Böhlke, 1984 in McCosker and Böhlke (1984); (Ophichthidae), String eel. Species last reported in the GOM in 1973 off the Florida coast and reported off the Atlantic coast in 2000, 2001 and 2004. Caires and Fonseca (2010) reported a mature female *Gordiichthysleibyi* from the stomach of a Yellowtail snapper (*Ocyuruschrysurus*) collected 113 km off the coast of Mucuri, Bahia, Brazil in 2005. Vouchered specimens reported in GBIF and FISHNET2 were caught in the Atlantic Ocean (off of North Carolina), not the Gulf or Caribbean Region, possibly due to being carried by the Gulf Stream. Status listed as “Data Deficient” on the IUCN Red List (McCosker 2015).

*Ophichthusomorgmus* McCosker and Böhlke, 1984 in McCosker and Böhlke (1984); (Ophichthidae), Dottedline snake eel. Last reported in 1999 off the Florida Keys. Status listed as “Data Deficient” on the IUCN Red List (Collette et al. 2022).

*Ophichthusrex* Böhlke and Caruso, 1980 in Böhlke and Caruso (1980); (Ophichthidae), Kingsnake eel. Status listed as “Least Concern” on the IUCN Red List (McCosker 2015).

Fig. 4

CLUPEIFORMES

Alosidae

*Alosaalabamae* Jordan and Evermann, 1896 - in Jordan and Evermann (1896); (Alosidae), Alabama shad. Status listed as “Near Threatened” on the IUCN Red List (Anonymous 2021).

*Alosachrysochloris* (Rafinesque, 1820) in Rafinesque (1820); (Alosidae), Skipjack shad. Status listed as “Least concern” on the IUCN Red List (Robertson and Caruso 2018).

*Brevoortiagunteri* Hildebrand, 1948 in Hildebrand (1948); (Alosidae), Finescale menhaden. Status listed as “Least concern” on the IUCN Red List (Collette et al. 2019).

*Brevoortiapatronus* Goode, 1878 in Goode (1878); (Alosidae), Gulf menhaden. Status listed as “Least concern” on the IUCN Red List (Collette et al. 2019).

*Neoopisthopteruscubanus* Hildebrand, 1948 in Hildebrand (1948); (Pristigasteridae), Cuban Longfin herring. Species last reported in 1937. Status listed as “Vulnerable” on the IUCN Red List (Caruso et al. 2018).

STOMIIFORMES

*Eustomiasleptobolus* Regan and Trewavas, 1930 in Regan and Trewavas (1930); (Stomiidae), Black dragonfish. The species was last reported in the GOM in 1960 off the coast of Louisiana and the species status is listed as “ Data Deficient” by IUCN (Harold and Milligan 2019).

Fig. 5

NEOTELEOSTS

Ateleopidae

*Ijimaiaantillarum* Howell Rivero, 1935 in Howell Rivero (1935); (Ateleopodidae), Jellynose. Species last reported in GOM in 2004. A material sample of *J.antillarum* is reported in GBIF from 2011 off the coast of Belize. Syverson et al. (2014) reported observations of *J.antillarum* near Roatán, Honduras between 2012 and 2015. Status listed as “Least Concern” on the IUCN Red List (Roa-Varón and Iwamoto 2019).

Aulopiformes

*Stemonosudisbullisi* Rofen, 1963 in Rofen (1963); (Paralepididae). Species was last collected in 2007 off the coast of Alabama. Status listed as “Data Deficient” on the IUCN Red List (Russell 2010).

Gadoidei

*Coryphaenoidesmexicanus* (Parr, 1946) in Parr (1946); (Macrouridae), Mexican grenadier. Status listed as “Least concern” on the IUCN Red List (Roa-Varón and Iwamoto 2019).

Fig. 6

PERCOMORPHA

ACANTHURIFORMES

Ogcocephalidae

*Halieutichthysintermedius* Ho, Chakrabarty and Sparks, 2010 in Ho et al. (2010); (Ogcocephalidae), Louisiana Pancake batfish. Species collected three times in October, 2010 following DWH. Status listed as “Least Concern” on the IUCN Red List (Collette et al. 2019).

*Ogcocephaluspantostictus* Bradbury, 1980 in Bradbury (1980); (Ogcocephalidae), Spotted batfish. Status listed as “Least Concern” on the IUCN Red List (Collette et al. 2015).

Oneirodidae

*Oneirodesbradburyae* Grey, 1957 in Grey (1957); (Oneirodidae), American Dreamer anglerfish. Collected once in 1954 off the coast of western Florida. A genetic sample was recorded in 2015 off the coast of Alabama by the DEEPEND Consortium (Schmutz 2022). Status listed as “Data Deficient” on the IUCN Red List (Carpenter et al. 2019).

Sciaenidae

*Cynoscionarenarius* Ginsburg, 1930 in Ginsburg (1930); (Sciaenidae), Sand seatrout. Status listed as “Least Concern” on the IUCN Red List (Espinosa-Perez and Robertson 2020).

Fig. 7

Sparidae

*Calamusarctifrons* Goode and Bean, 1882 in *Goode and Bean (1882)*; (Sparidae), Grass porgy. Status listed as “Least concern” on the IUCN Red List (Collette et al. 2019).

*Calamuscampechanus* Randall and Caldwell, 1966 in Randall and Caldwell (1966) (Sparidae), Campeche porgy. Last reported on GBIF in 2007, although Poot‐López et al. (2017) reported 15 *C.campechanus* caught between Sept 2015 and October 2016 off the northern coast of the Yucatan Peninsula, Mexico. Borges-Ramirez et al. (2020) reported 40 *C.campechanus* caught in 2019 in Los Petenes Biosphere Reserve, Campeche Bay, Mexico. Status listed as “Data Deficient” on the IUCN Red List (Collette et al. 2019).

Tetraodontidae

*Sphoeroidesparvus* Shipp and Yerger, 1969 in Shipp and Yerger (1969); (Tetraodontidae). Least puffer. Status listed as “Least Concern” on the IUCN Red List (Anonymous 2014).

*Sphoeroidesspengleri* (Bloch, 1785) in Bloch (1785); (Tetraodontidae), Bandtail puffer. Status listed as “Least Concern” on the IUCN Red List (Shao et al. 2014).

Fig. 8

ATHERINIFORMES

Atherinopsidae

*Atherinellaschultzi* (Álvarez and Carranza, 1952) Álvarez del Villar and Carranza (1952); (Atherinopsidae), Chimalapa silverside. Status listed as “Data Deficient” on the IUCN Red List (Espinosa Pérez and Lambarri Martínez 2019).

*Menidiaclarkhubbsi* Echelle and Mosier, 1982 in Echelle and Mosier (1982) (Atherinopsidae), Texas silverside. Species last reported in 2000 off the Texas coast. Status listed as “Data Deficient” on the IUCN Red List (Collette et al. 2019).

*Menidiacolei* Hubbs, 1936 in Hubbs (1936); (Atherinopsidae), Golden silverside. Species was last reported in 2009. Status listed as “Vulnerable” on the IUCN Red List (Schmitter-Soto et al. 2019).

*Menidiaconchorum* Hildebrand and Ginsburg, 1927 in Hildebrand and Ginsburg (1927); (Atherinopsidae), Key silverside. Species was collected twice in 2019 of the Florida Keys and observed twice (2011, 2014) in the Cayman Islands by divers. Status listed as “Endangered” on the IUCN Red List (Collette et al. 2015).

Cyprinodontidae

*Floridichthyscarpio* (Günther, 1866) in Günther (1866); (Cyprinodontidae), Goldspotted killifish. Status listed as “Least Concern” on the IUCN Red List (Chao et al. 2015).

*Jordanellafloridae* Goode and Bean, 1879 in Goode and Bean (1879); (Cyprinodontidae), American flagfish. Status is not listed by the IUCN Red List.

*Jordanellapulchra* (Hubbs, 1936) in Hubbs (1936); (Cyprinodontidae), Progreso or Yucatan flagfish. Status listed as “Least Concern” on the IUCN Red List (Schmitter-Soto and Vega-Cendejas 2019).

Fig. 9

Fundulidae

*Fundulusgrandis* Baird and Girard, 1853 in Baird and Girard (1853); (Fundulidae), Gulf killifish. Status listed as “Least Concern” on the IUCN Red List (Collette et al. 2019).

*Fundulusjenkinsi* (Evermann, 1892)in Evermann (1892); (Fundulidae), Saltmarsh topminnow. Status listed as “Vulnerable” on the IUCN Red List (Collette et al. 2019).

*Funduluspersimilis* Miller, 1955 in Miller (1955); (Fundulidae), Yucatan killifish. Species last reported in 2005 off the Yucatán Peninsula, Mexico. Status listed as “Endangered” on the IUCN Red List (Jelks et al. 2019).

*Funduluspulvereus* (Everman, 1892) in Evermann (1892); (Fundulidae), Bayou killifish. Status listed as “Least Concern” on the IUCN Red List (Collette et al. 2019).

*Fundulusxenica* Jordan and Gilbert, 1882 (formerly *Adiniaxenica*) in Jordan and Gilbert (1882); (Fundulidae), Diamond killifish. Status listed as “Least Concern” on the IUCN Red List (Anonymous 2015).

Poeciliidae

*Gambusiayucatana* Regan, 1914 in Regan (1914); (Poeciliidae), Yucatan gambusia. Species last reported on FishNet2 in 2010 in the Yucatán Peninsula, Mexico. Rodríguez-Fuentes et al. (2016) reported collecting *G.yucatana* (n = 38) in the Yucatan Peninsula wetlands during a monitoring campaign in May 2014. Aguilar et al. (2021) reported collecting juvenile *G.yucatana* (number not reported) from a small stream in San Francisco de Campeche City, Mexico in 2017. Aguilar et al. (2022) reported collecting *G.yucatana* (number not reported) from a small stream in San Francisco de Campeche City, Mexico in 2020. Status listed as “Least Concern” on the IUCN Red List (Collette et al. 2019).

Fig. 10

BATRACHOIDIDAE

*Opsanuspardus* (Goode and Bean, 1880) in Goode and Bean (1880); (Batrachoididae), Leopard toadfish. Status listed as “Least Concern” on the IUCN Red List (Polanco Fernandez et al. 2015).

*Sanopusreticulatus* Collette, 1983 in Collette 1983); (Batrachoididae), Reticulated toadfish. Observed once in 2015 off the Yucatan Peninsula in Mexico. Status listed as “Endangered” on the IUCN Red List (Collette et al. 2015).

Fig. 11

BLENNIFORMES

*Chasmodeslongimaxilla* Williams, 1983 in Williams (1983); (Blenniidae), Stretchjaw blenny. Status listed as “Least concern” on the IUCN Red List (Anonymous 2014).

*Hypleurochiluscaudovittatus* Bath, 1994 in Bath (1994); (Blenniidae), Zebratail blenny. Last reported on FishNet2 in 2004. Schrandt (2018) reported collecting *H.caudovittatus* in the Big Bend Region of Florida during 2008-2015 trawls. Munnelly et al. (2021) reported possible sightings of *H.caudovittatus* during 2013–2014 from remote video and diver surveys around 150 small oil platforms in nearshore federal waters off different points of the Louisiana coast at ≤ 18 m depth. Status listed as “Least Concern” on the IUCN Red List (Anonymous 2014).

*Hypleurochilusmultifilis* (Girard, 1858) in Girard (1858); (Blenniidae), Featherduster blenny. Status listed as “Least Concern” on the IUCN Red List (Smith-Vaniz et al. 2014).

*Lupinoblenniusnicholsi* (Tavolga, 1954) in Tavolga (1954); (Blenniidae), Highfin blenny. Turner et al. (2022) reported range expansion of *L.nicholsi* to the northern GOM and collected a single specimen in 2021 off Dauphin Island, Alabama. Lima et al. (2015) reported *L.nicholsi* as the most abundant larvae sampled during trawling in 2012/13 along a floodplain across the north-eastern coast of Brazil. Status listed as “Least Concern” on the IUCN Red List (Smith-Vaniz et al. 2014).

Fig. 12

CARANGIFORMES

*Citharichthysabbotti* Dawson, 1969 in Dawson (1969); (Cyclopsettidae), Veracruz whiff. Species last reported in 2001 off the Texas coast. Status listed as “Least concern” on the IUCN Red List (Munroe et al. 2015).

*Gymnachirustexae* (Gunter, 1936) in Gunter (1936); (Achiridae), Gulf of Mexico Fringed sole. Status listed as “Least Concern” on the IUCN Red List (Tornabene et al. 2015).

*Trichopsettaventralis* (Goode and Bean, 1885) in Goode and Bean (1885); (Bothidae), Sash flounder. Status listed as “Least Concern” on the IUCN Red List (Anonymous 2021).

Fig. 13

GOBIIFORMES

Gobiidae

*Bollmanniacommunis* Ginsburg, 1942 in Ginsburg (1942); (Gobiidae), Ragged goby. Status listed as “Least concern” on the IUCN Red List (Pezold et al. 2015).

*Bollmanniaeigenmanni* (Garman, 1896) Garman (1896); (Gobiidae), Shelf goby. Status listed as “Least concern” on the IUCN Red List (Pezold et al. 2015).

*Coryphopteruspunctipectophorus* Springer, 1960 in Springer (1960); (Gobiidae), Spotted goby. Status listed as “Least concern” on the IUCN Red List (Pezold et al. 2015).

*Ctenogobiusclaytonii* (Meek, 1902) in Meek (1902); (Gobiidae), Black fin goby. The species was last reported off the Yucatan Peninsula, Mexico in 2015. Status is listed as “Vulnerable” on the IUCN Red List (Pezold 2019).

*Gobiosomalongipala* Ginsburg, 1933 in Ginsburg (1933); (Gobiidae), Twoscale goby. Status listed as “Least Concern” on the IUCN Red List (Anonymous 2015).

*Varicusbenthonis* (Ginsburg, 1953) formerly *Chriolepisbenthonis* in Ginsburg (1953); (Gobiidae), Deepwater goby. There are five reports of this species of GBIF collected off the Yucatan Peninsula, Mexico with no date given. Status listed as “Data Deficient” on the IUCN Red List (Pezold et al. 2015).

*Varicusmarilynae* Gilmore, 1979 in Gilmore (1979); (Gobiidae), Orange belly goby. The species was last reported off the coast of Florida in 1974. Status listed as “Data Deficient” on the IUCN Red List (Gilmore et al. 2015).

*Varicusvespa* (Hastings and Bortone, 1981) formerly *Chriolepisvespa* in Hastings and Bortone (1981); (Gobiidae), Wasp goby. The species was last reported off the coast of Florida in 2006. Status listed as “Least Concern” on the IUCN Red List (Pezold et al. 2015).

Microdesmidae

*Microdesmuslanceolatus* Dawson, 1962 in Dawson (1962); (Microdesmidae), Lancetail wormfish. Species last reported in 1994. Status listed as “Least Concern” on the IUCN Red List (Collette et al. 2015).

LABRIFORMES

*Halichoeresburekae* Weaver and Rocha, 2007 in Weaver and Rocha (2007); (Labridae), Mardi Gras wrasse. Species listed as “Endangered” on the IUCN Red List (UICN, 2022). Robertson et al. (2016) reported *H.burekae* to be abundant in the Cayo Arcas reefs off the Yucatan Peninsula in Mexico. Francisco and Fraco-Mej (2017) reported *H.burekae* as one of the most abundant species encountered at Enmedio Reef off Veracruz, Mexico. Escarcega-Quiroga and Flores-Serrano (2020) reported *H.burekae* to be a frequently consumed species in the diet of the introduced Red lionfish (*Pteroisvolitans*) in coral reefs of northern Veracruz, Mexico. Gonzlez-Gandara and Chavez (2020) reported the relative abundance of *H.burekae* as > 30% in Veracruz Coast, 2-5% in Campeche Bank and < 1 in the Mexican Caribbean. Status listed as “Endangered” on the IUCN Red List (Rocha et al. 2015).

Fig. 14

OPHIDIIFORMES

Bythitidae

*Parasaccogasterrhamphidognatha* (Cohen, 1987) in Cohen (1987); (Bythitidae). Collected once in 1969 off the coast of Alabama. Status listed as “Data Deficient” on the IUCN Red List (Cobián Rojas et al. 2019).

Dinematichthyidae

*Gunterichthyslongipenis* Dawson, 1966 in Dawson (1966); (Dinematichthyidae), Gold brotula. Observed twice in 2011 off the coast of southern Florida. Status listed as “Least Concern” on the IUCN Red List (Anonymous 2015).

*Ogilbiacayorum* Evermann and Kendall, 1898 in Evermann and Kendall (1898); (Dinematichthyidae), Key brotula. Status listed as “Least Concern” on the IUCN Red List (Carpenter and Robins 2015).

PERCIFORMES

Triglidae

*Prionotuslongispinosus* Teague, 1951 in Teague (1951); (Triglidae), Bigeye searobin. Status listed as “Least Concern” on the IUCN Red List (Collette et al. 2015).

*Prionotusmartis* Ginsburg, 1950 in Ginsburg (1950); (Triglidae), Gulf of Mexico Barred searobin. Status listed as “Least Concern” on the IUCN Red List (Collette et al. 2015).

*Prionotusparalatus* Ginsburg, 1950 in Ginsburg (1950); (Triglidae), Mexican searobin. Status listed as “Least Concern” on the IUCN Red List (Collette et al. 2015).

Zoarcidae

*Exechodontesdaidaleus* DeWitt, 1977 in DeWitt (1977); (Zoarcidae), Outwordtoothed eelpout. The species was last reported in the GOM in 2007 off the coast of Florida. Status listed as “Least Concern” on the IUCN Red List (Vega-Cendejas et al. 2019).

*Lycenchelysbullisi* Cohen, 1964 in Cohen (1964); (Zoarcidae), Gulf eelpout. Species last reported in 1999 off of the Florida Keys. Status listed as “Least Concern” on the IUCN Red List (Cobián Rojas et al. 2019).

Fig. 15

SCOMBRIFORMES

*Hyperoglyphebythites* (Ginsburg, 1954) in Ginsburg (1954); (Centrolophidae), Black driftfish. Observed once in 2019 off the coast of Texas. Status listed as “Least Concern” on the IUCN Red List (Collette et al. 2015).

SYNGNATHIFORMES

*Syngnathustexanus* Gilbert, 2013 in Gilbert et al. (2013); (Syngnathidae), Texas pipefish. The species was last observed in 1983. Status is not assessed by the IUCN Red List.

Fig. 16

## Discussion

Understanding the impacts of catastrophic environmental events such as the 2010 Gulf of Mexico Oil Spill does not end when the wellhead is capped or when the last drops of oil cease to flow. The disaster only begins to end when the data no longer show impacts of the event. We are far from the beginning of the end for DWH. Lingering chemicals, lost generations of wildlife and a continued ecosystem imbalance may all be factors that prevent an environment from rebounding from such cataclysmic events (Turner and Rabalais 2019). However, the environment's ability to recover should also not be overlooked. This paper is the third in a series looking at distributions of endemic and threatened fishes that have distributions in the region of the 2010 Oil Spill. The first paper (Chakrabarty et al. 2012) mapped the surface oil slick from DWH and the distribution of fish species known to have specimens collected in the region; the second (Chakrabarty et al. 2016) re-examined those species to look for evidence of a continued impact; and here again we do a longer term (10 year) review of the populations of these species that may have been most impacted by DWH. Different from those previous studies which exclusively used museum voucher (reference specimens that are identified and catalogued in natural history collections) as the only evidence of a species existence or persistence in an area, this paper also included a literature search and other evidence that a species may be known from an area - even without a museum voucher. The need for museum vouchers as definitive evidence of a species existence in a given time and place should not be underestimated (Buckner et al. 2021), but we also recognise that qualified individuals can also make visual identifications that can be useful in expanding our knowledge of species distributions. GBIF recently added observational data (in the absence of a voucher) as part of its search tool, a feature not available for Chakrabarty et al. (2016), Chakrabarty et al. (2012)Chakrabarty et al. 2016) which also relied on GBIF. To acknowledge that the observational data do not have the same weight as a physical specimen (i.e. it is difficult to check for misidentifications when a physical specimen is missing (Chakrabarty 2010), we separate those data in the results above. The addition of FishNet2, which is a database specific to fish collections, shows how there can be discrepancies even between partnering databases (much of the FishNet2 data are often uploaded to GBIF) (note that, in some of the graphs, the symbols may obscure each other and the FishNet2 and GBIF data were typically the same). At the time of writing, we are more than 13 years past the period of the spill, but it can take many years for the data to be merged and uploaded between individual databases. For that reason, we focused our review for the time up to 10 years post-DWH.

Chakrabarty et al. (2012) reported that “Endemic species of greatest concern” which were species potentially impacted the most by the oil spill because greater than 35% of their historical records were from the spill zone: The species with the highest level of distribution overlap were, from highest to lowest: *Parasaccogasterrhamphidognatha* (formerly *Saccogasterrhamphidognatha*) (100%), *Oneirodesbradburyae* (100%), *Etmopterusschultzi* (90%), *Gunterichthyslongipenis* (88%), *Hyperoglyphebythites* (82%), *Ophichthusrex* (82%), *Dipturusoregoni* (80%), *Springeriafolirostris* (formerly *Anacanthobatisfolirostris*) (79%), *Halieutichthysintermedius* (68%), *Bollmanniaeigenmanni* (64%), *Coryphaenoidesmexicanus* (54%), *Eptatretusspringeri* (54%), *Leucorajalentiginosa* (53%), *Lycenchelysbullisi* (50%), *Prionotuslongispinosus* (50%), *Microdesmuslanceolatus* (43%), *Mustelussinusmexicanus* (43%), *Bollmanniacommunis* (41%), *Eustomiasleptobolus* (40%) and *Opsanuspardus* (39%). One quarter of all endemics to the Gulf of Mexico were in this highest potential impact category reported by Chakrabarty et al. (2012).

Of these species of greatest concern, six remain unsampled: *Eustomiasleptobolus*, *Etmopterusschultzi*, *Lycenchelysbullisi*, *Microdesmuslanceolatus*, *Neoopisthopteruscubanus* and *Parasaccogasterrhamphidognatha* (see Table 1). Notably, of these species, some have not been seen since their first description many years before the spill. It would be disingenuous to link their absence in the Gulf in recent years to DWH when many were “missing” from well before that time. However, the opportunity to rediscover these species while also monitoring the Gulf post-spill should not be lost.

Chakrabarty et al. (2012) also labelled “Endemic species of concern” as species with less than 35% of historical records being from the spill zone (here called species of “lesser concern”). These included *Trichopsettaventralis* (31%), *Dipturusolseni* (29%), *Hypleurochilusmultifilis* (25%), *Eptatretusminor* (23%), *Funduluspulvereus* (18%), *Gymnachirustexae* (16%), *Adiniaxenica* (13%), *Fundulusgrandis* (13%), *Cynoscionarenarius* (12%), *Rajatexana* (11%), *Brevoortiapatronus* (11%), *Ijimaiaantillarum* (8%), *Prionotusmartis* (5%), *Fundulusjenkinsi* (4%), *Ogcocephaluspantostictus* (3%), *Brevoortiagunteri* (2%), *Alosachrysochloris* (2%), *Alosaalabamae* (1%), *Sphoeroidesspengleri* (0.4%) and *Lepisosteusoculatus* (0.2%) and here we also consider those with no overlap with the spill, but that were recognised Gulf endemics. Several of these have yet to be collected post-spill or have only been observed (Table 2).

Notably, the newly described Pocket Shark, *Mollisquamamississippiensis*, (Grace et al. 2019), the newest endemic for the Gulf Of Mexico, is only known from a single specimen, the holotype, collected in 2010. Several “missing” species reported in Chakrabarty et al. (2016) have now been observed or collected (see Table 3). Adding observations and conducting a literature search for species that had not been observed since 2010 has reduced the number of missing species; however, it should be noted that species only observed should still be considered imperilled until direct evidence (e.g. a voucher or a positive photo identification with location data can verify these results).

Although several endemic species of concern remain “missing” and the lack of samples may not be necessarily connected to the 2010 Oil Spill, their absence remains telling given how increased sampling efforts specifically looking at post-spill fish distributions, including GoMRI (the *Gulf of Mexico Research Initiative* https://gulfresearchinitiative.org/; Murawski et al. (2023)). However, the efforts of those and other groups are yet to be fully included in global databases, such as FishNet2 and GBIF.

The Gulf of Mexico continues to face many challenges from the Dead Zone, to climate change, loss of coast habitats and continued oil spills (Turner and Rabalais 2019). Efforts like this report aim to bring attention to vulnerable species that continue to be impacted by human activities and to the unique endemic fauna of the region.

## Figures and Tables

**Figure 1.**
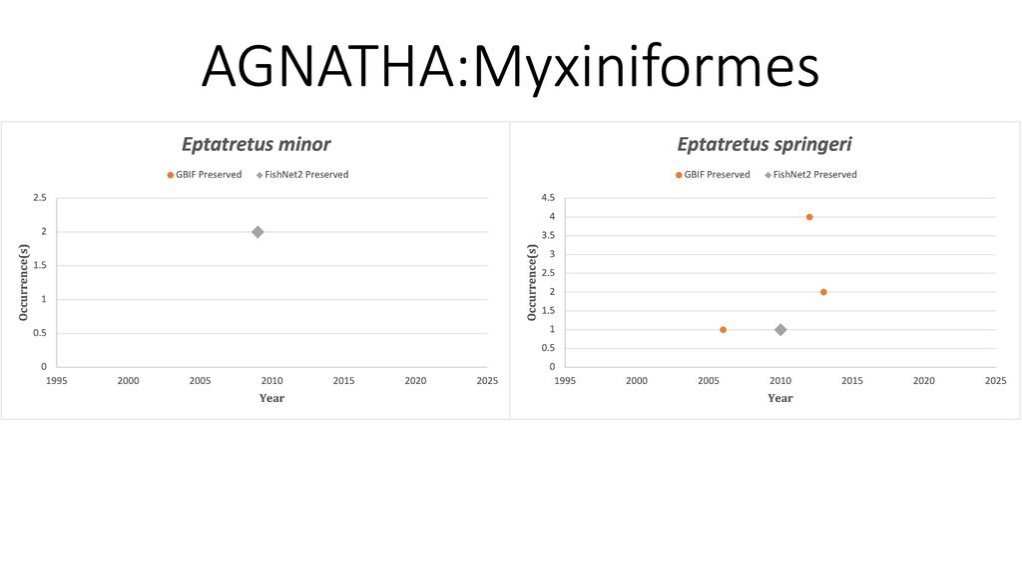
Figure 1: Collections and observation data for species of Agnatha.

**Figure 2.**
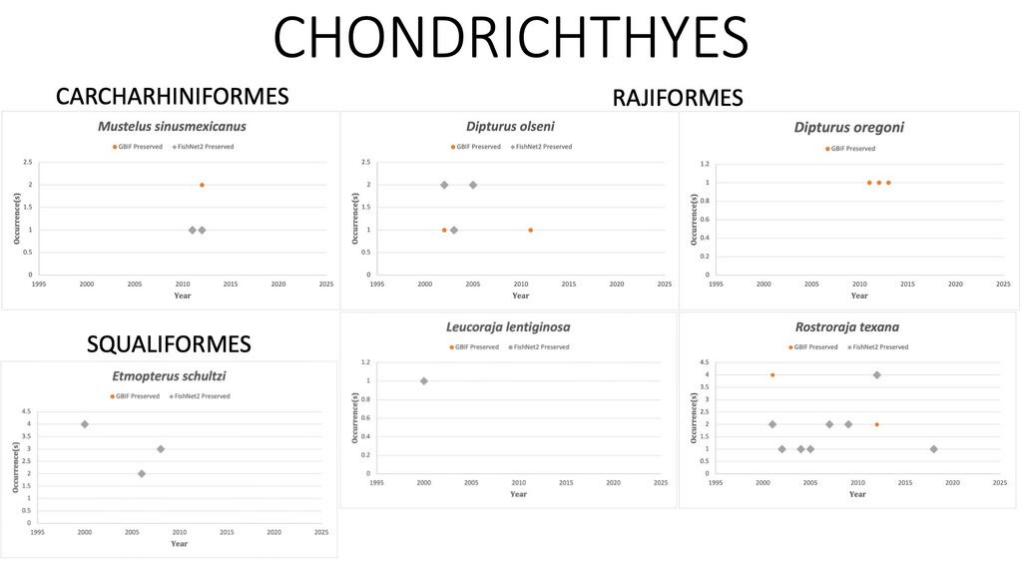
Figure 2: Collections and observation data for species of Chondrichthyes.

**Figure 3.**
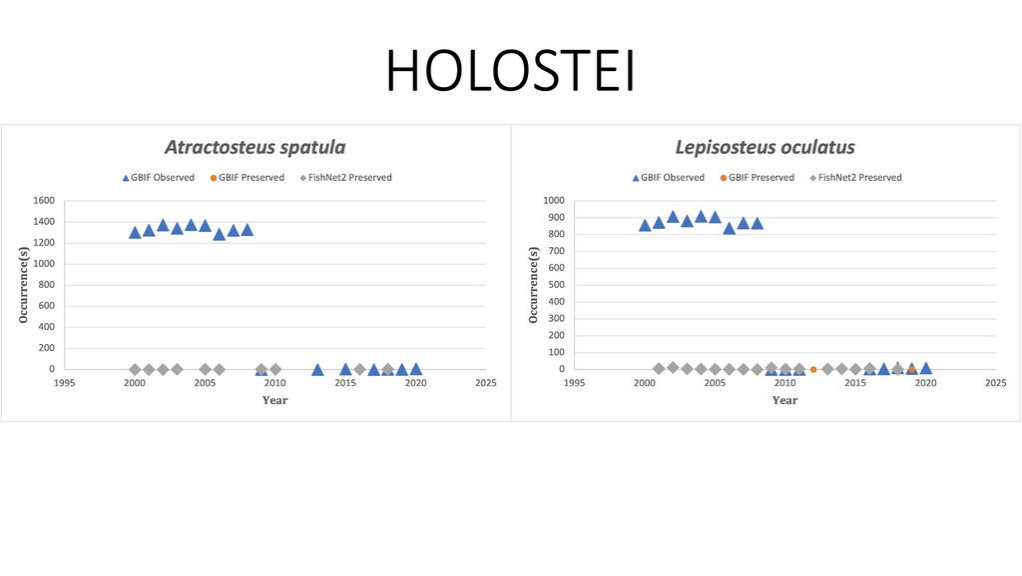
Figure 3. Collections and observation data for species of Holostei.

**Figure 4.**
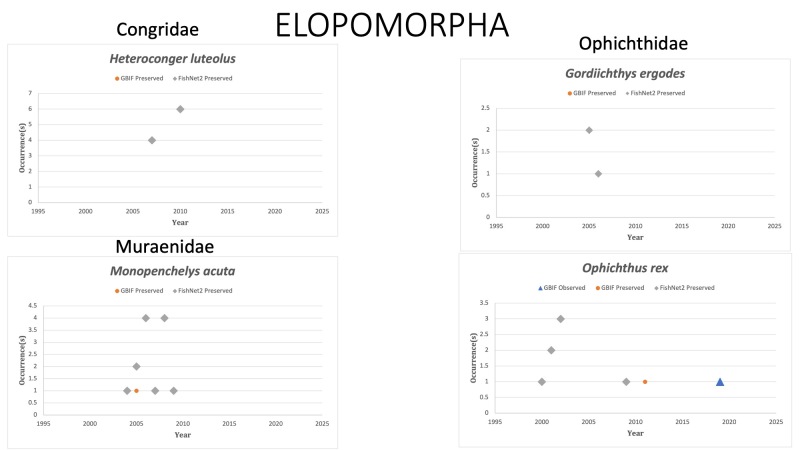
Figure 4. Collections and observation data for species of Elopomorpha.

**Figure 5.**
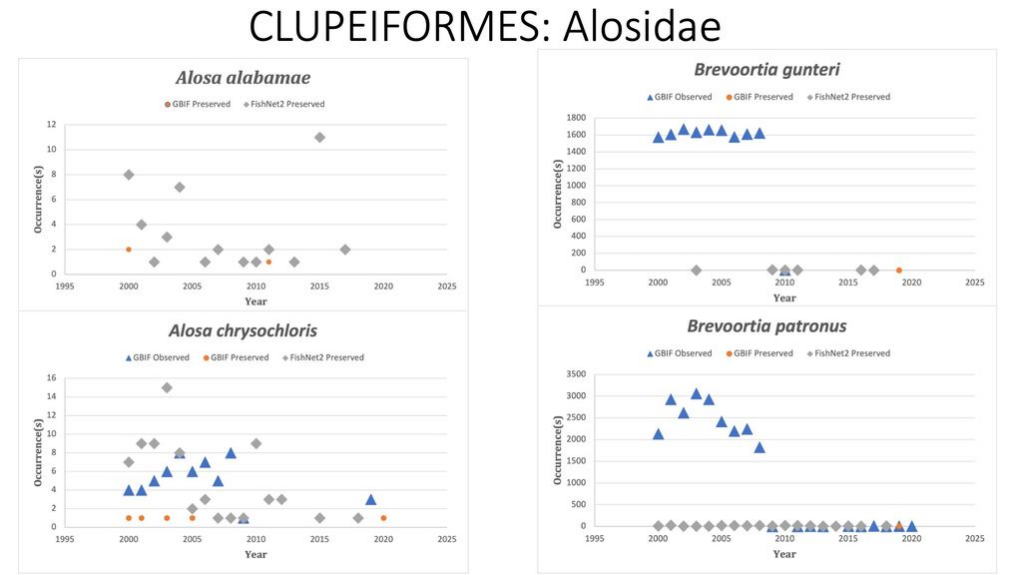
Figure 5: Collections and observation data for species of Clupeiformes.

**Figure 6.**
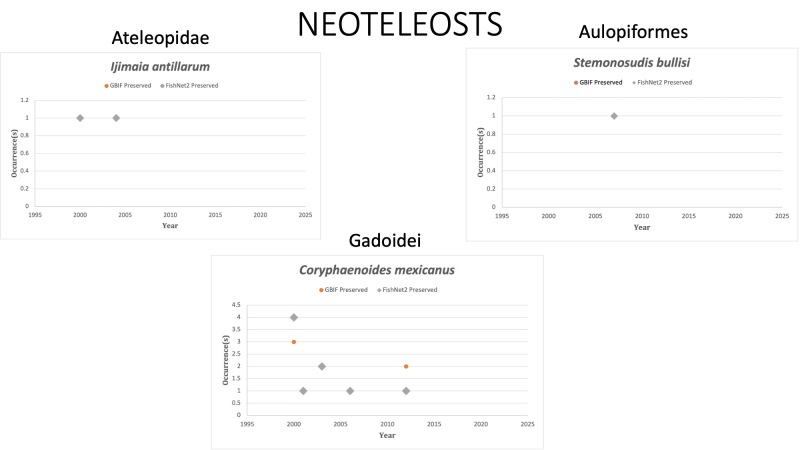
Figure 6: Collections and observation data for species of Neoteleosts (non-Percomorpha).

**Figure 7.**
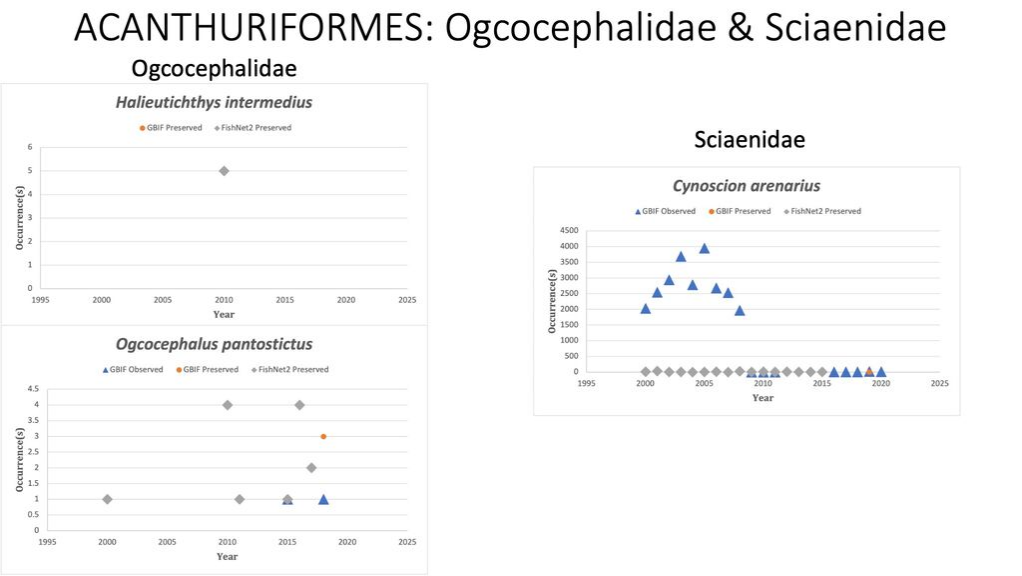
Figure 7: Collections and observation data for species of Acanthuriformes in Ogcocephalidae and Sciaenidae.

**Figure 8.**
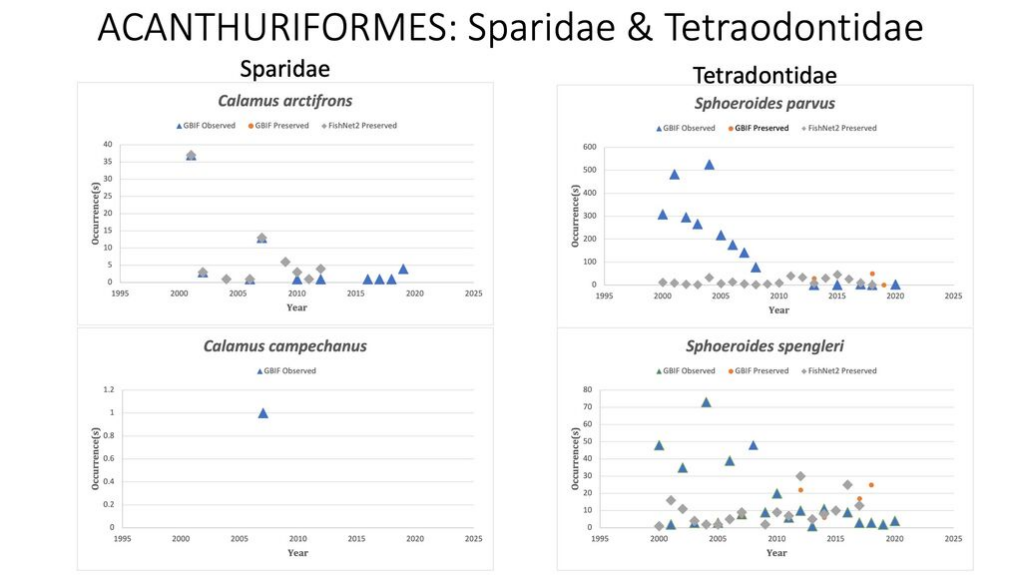
Figure 8: Collections and observation data for species of Acanthuriformes in Sparidae and Tetraodontidae.

**Figure 9.**
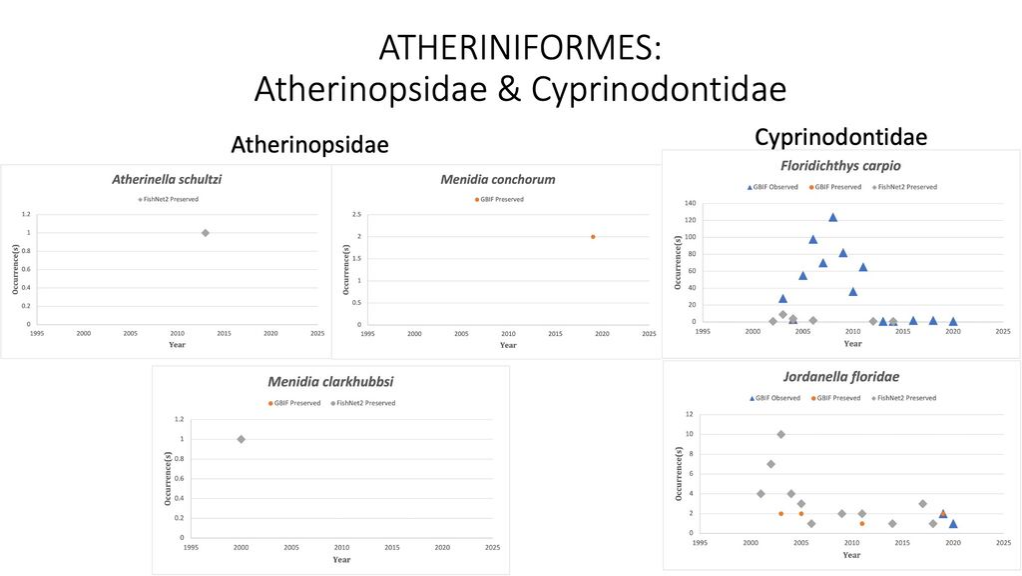
Figure 9: Collections and observation data for species of Atheriniformes in Atherinopsidae (left) and Cyprinodontidae (right).

**Figure 10.**
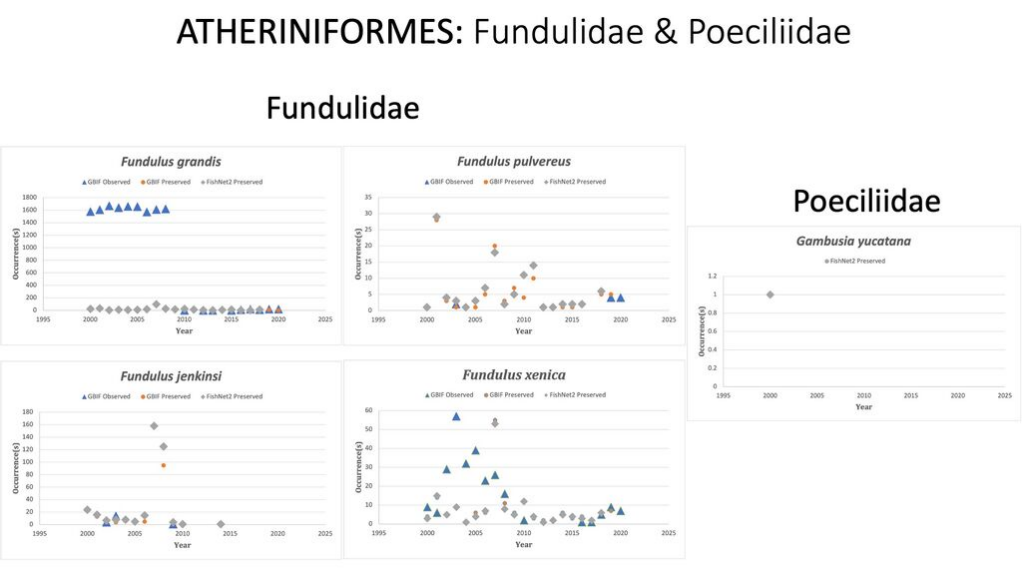
Figure 10: Collections and observation data for species of Atheriniformes in Fundulidae and Poeciliidae.

**Figure 11.**
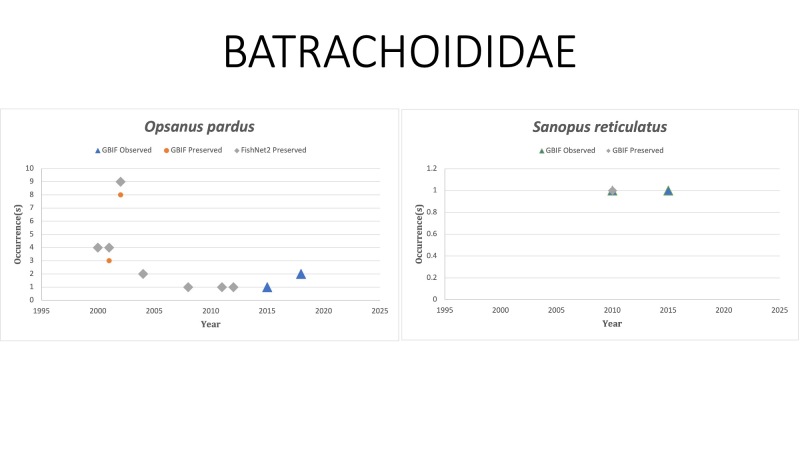
Figure 11: Collections and observation data for species of Batrachoididae.

**Figure 12.**
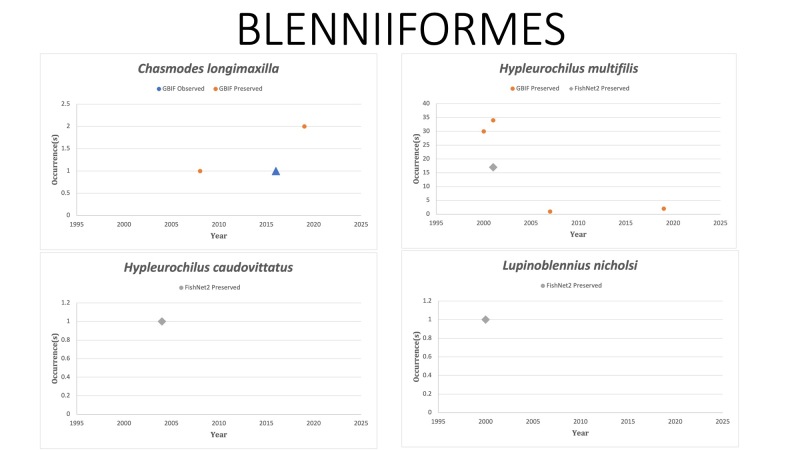
Figure 12: Collections and observation data for species of Blenniiformes.

**Figure 13.**
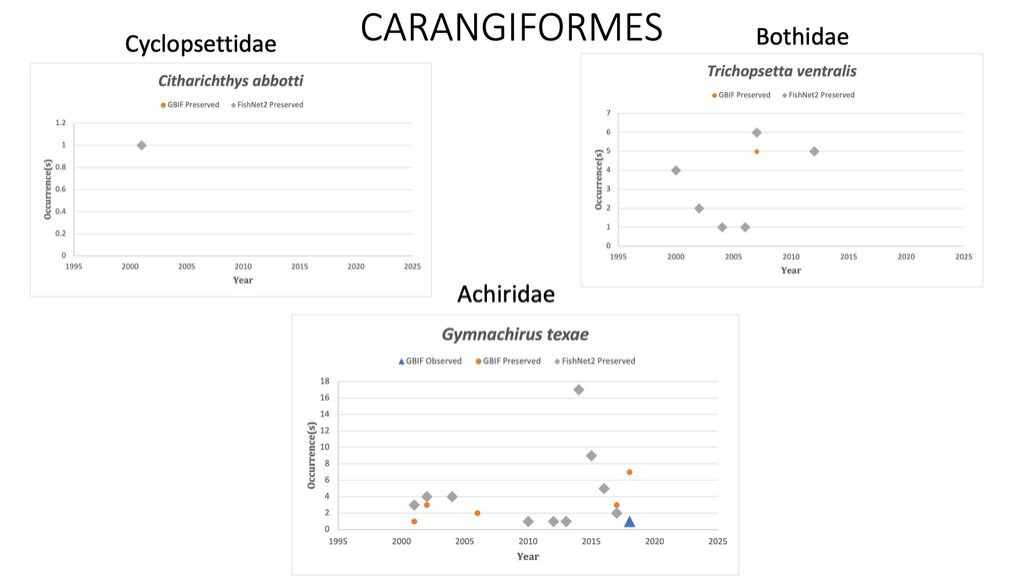
Figure 13: Collections and observation data for species of Carangiformes.

**Figure 14.**
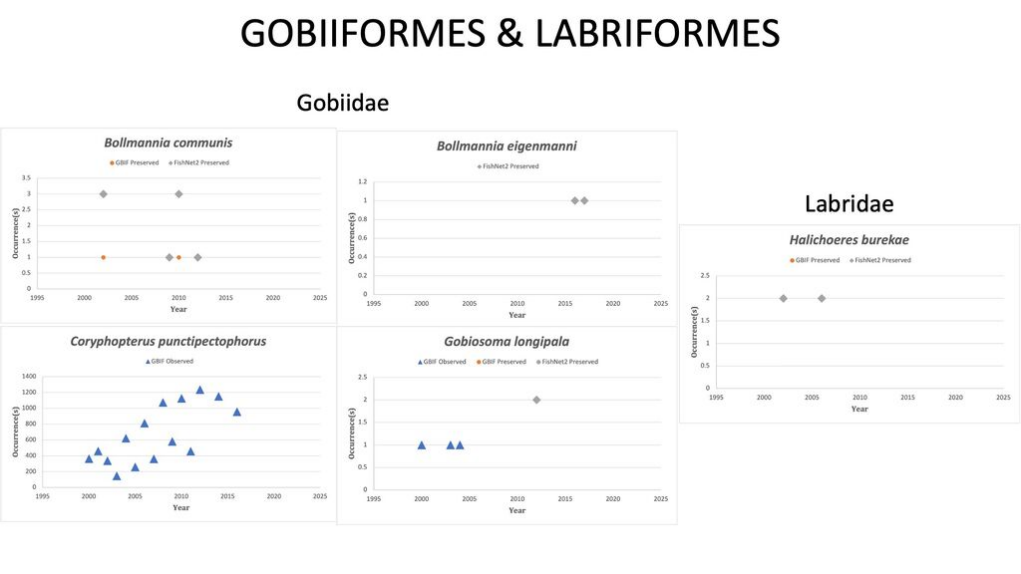
Figure 14: Collections and observation data for species of Gobiiformes and Labriformes.

**Figure 15.**
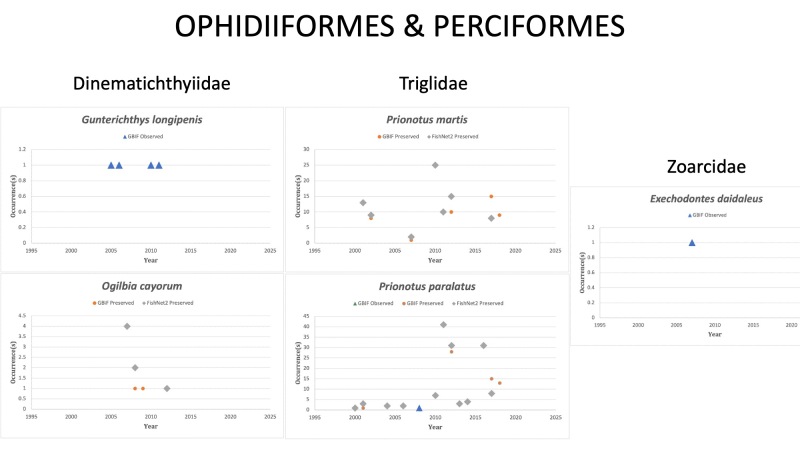
Figure 15: Collections and observation data for species of Ophidiiformes and Perciformes.

**Figure 16.**
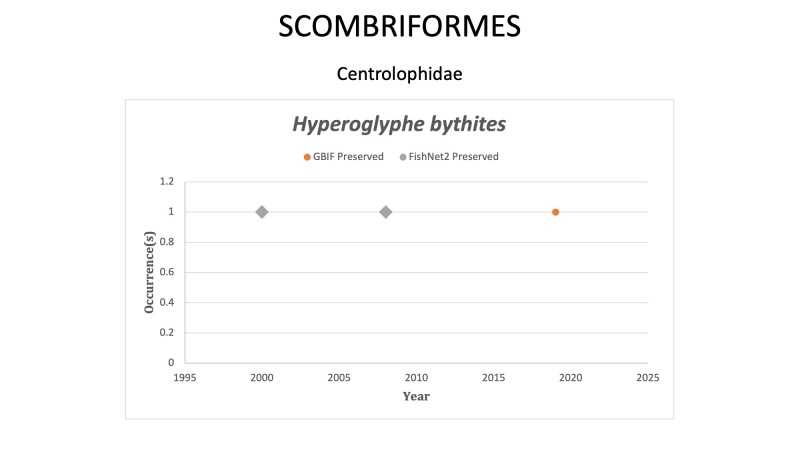
Figure 16: Collections and observation data for species of Scombriformes.

**Table 1.** Species of Greatest Concern not collected or observed since the DWH (and their last identification date).

**Species of Greatest Concern from the** **Gulf of Mexico**	**Last Identification Date**
*Etmopterusschultzi* Bigelow, Schroeder & Springer 1953. (Squaliformes, Etmopteridae)	2009
*Lycenchelysbullisi* Cohen 1964. (Perciformes, Zoarcidae)	1999
*Microdesmuslanceolatus* Dawson 1962 (Gobiiformes, Microdesmidae)	1994
*Parasaccogasterrhamphidognatha* (Cohen 1987) (Ophidiiformes, Bythitidae)	1969
*Springeriafolirostris* Bigelow and Schroeder 1951 (Rajiforms, Anacanthobatidae)	2004

**Table 2.** Endemic species of Lesser Concern (less than 35% of their distribution was noted to be in the region of the spill) not collected in the GOM following DWH (and their last identification date).

**Missing Gulf of Mexico Endemics**	**Last Seen/Collected**
*Eptatretusminor* (Fernholm & Hubbs 1981); (Myxiniformes, Myxinidae)	2009
*Exechodontesdaidaleus* DeWitt 1977; (Scorpaeniformes, Zoarcidae)	2007
*Funduluspersimilis* (Miller 1955); (Atheriniformes, Fundulidae)	2005
*Gordiichthysergodes* McCosker, Böhlke & Böhlke 1989; (Elopomorpha, Ophichthidae)	2006
*Gordiichthysleibyi* McCosker & Böhlke 1984; (Elopomorpha, Ophichthidae)	2005
Gunterichthyslongipenis* Dawson 1966; (Ophidiiformes, Dinematichthyidae)	observed 2011
Hyperoglyphebythites* (Ginsburg 1954); (Scombriformes, Centrolophidae)	observed in 2019
Jordanellapulchra* (Hubbs 1936); (Atheriniformes, Cyprinodontidae)	observed in 2019
Leucorajalentiginosa* (Bigelow & Schroeder 1951); (Rajiformes, Rajidae)	observed in 2012
*Menidiaclarkhubbsi* Echelle & Mosier 1982; (Atheriniformes, Atherinopsidae)	2000
*Menidiacolei* Hubbs 1936; (Atheriniformes, Atherinopsidae)	2009
*Mollisquamamississippiensis* Grace, Doosey, Denton, Naylor, Bart & Maisey 2019. (Squaliformes, Dalatiidae)	2010
*Monopenchelysacuta* (Parr 1930); (Elopomorpha, Muraenidae)	2007
*Neoopisthopteruscubanus* Hildebrand 1948; (Clupeiformes, Pristigasteridae)	1937
*Ophichthusomorgmus* McCosker & Böhlke 1984; (Elopomorpha, Ophichthidae)	1999
*Parmaturuscampechiensis* Springer 1979; (Carcharhiniformes, Pentanchidae)	1970
Sanopusreticulatus* Collette 1983; (Actinopterygii, Batrachoididae)	observed 2015
*Stemonosudisbullisi* Rofen 1963; (Neoteleosts, Paralepididae)	2007
*Syngnathustexanus* Gilbert 2013; (Sygnathiformes, Syngnathidae)	1983
*Varicusbenthonis* (Ginsburg 1953); (Gobiiformes, Gobiidae)	no specific year available
*Varicusmarilynae* Gilmore 1979; (Gobiiformes, Gobiidae)	1974
*Varicusvespa* (Hastings & Bortone 1981); (Gobiiformes, Gobiidae)	2006

**Table 3.** Recently reported “found” (once “missing”; **Chakrabarty (2016))** species in the GOM following DWH and the number of reportings **(collected or observed).**

**Gulf of Mexico Species Once "Missing", Now Found**	**Last Collected/Reported**
*Calamuscampechanus* Randall & Caldwell 1966; (Acanthuriformes, Sparidae)	reported 2+ times since 2010
*Chasmodeslongimaxilla* Williams 1983; (Blenniformes, Blenniidae)	observed and collected 2 times since 2010
*Coryphopteruspunctipectophorus* Springer 1960; (Gobiiformes, Gobiidae)	observed more than 5 times since 2010
*Ctenogobiusclaytonia* (Meek 1902); (Gobiiformes, Gobiidae)	collected 1 time since 2010
*Dipturusolseni* (Bigelow & Schroeder 1951); (Rajiformes, Rajidae)	collected 1 time since 2010
*Dipturusoregoni* (Bigelow & Schroeder 1958); (Rajiformes, Rajidae)	collected 3 times since 2010
*Fundulusjenkinsi* (Evermann 1892); (Atheriniformes, Fundulidae)	collected 1 time since 2010
*Gunterichthyslongipenis* Dawson 1966; (Ophidiiformes, Dinematichthyidae)	observed 2 times since 2010
*Halichoeresburekae* Weaver & Rocha 2007; (Labriformes, Labridae)	observed and collected more than 50 times since 2010
*Heterocongerluteolus* Smith 1989; (Elopomorpha, Congridae)	observed and collected more than 5 times since 2010
*Hyperoglyphebythites* (Ginsburg 1954); (Scombriformes, Centrolophidae)	observed 1 time since 2010
*Hypleurochiluscaudovittatus* Bath 1994. (Blenniformes, Blenniidae)	reported 2 times since 2010
*Hypleurochilusmultifilis* (Girard 1858); (Blenniformes, Blenniidae)	collected 2 times since 2010
*Ijimaiaantillarum* Howell Rivero 1935; (Neoteoleost, Ateleopodidae)	observed 2 times since 2010
*Jordanellapulchra* (Hubbs 1936); (Atheriniformes, Cyprinodontidae)	observed 1 time since 2010
*Lupinoblenniusnicholsi* (Tavolga 1954); (Blenniformes, Blenniidae)	observed 1 times since 2010
*Menidiaconchorum* Hildebrand & Ginsburg 1927; (Atherinopsiformes, Atherinopsidae)	observed and collected 2 times since 2010
*Ogilbiacayorum* Evermann & Kendall 1898; (Ophidiiformes, Bythitidae)	observed and collected 2 times since 2010
*Oneirodesbradburyae* Grey 1957; (Acanthuriformes, Oneirodidae)	collected 1 time since 2010
*Ophichthusrex* Böhlke & Caruso 1980; (Elopomorpha, Ophichthidae)	observed and collected 2 times since 2010
*Sanopusreticulatus* Collette 1983; (Actinopterygii, Batrachoididae)	observed 1 time since 2010
